# Effects of applying ramie fiber nonwoven films on root-zone soil nutrient and bacterial community of rice seedlings for mechanical transplanting

**DOI:** 10.1038/s41598-020-60434-3

**Published:** 2020-02-26

**Authors:** Wanlai Zhou, Jing Chen, Zhiyong Qi, Chaoyun Wang, Zhijian Tan, Hongying Wang, Zhenxie Yi

**Affiliations:** 1 College of Agronomy, Hunan Agricultural University, Changsha, 410128 China; 2 Institute of Bast Fiber Crops, Chinese Academy of Agricultural Sciences, Changsha, 410205 China; 3 Institute of Urban Agriculture, Chinese Academy of Agricultural Sciences, Chengdu, 610213 China

**Keywords:** Agroecology, Plant ecology, Agroecology

## Abstract

Raising rice seedlings in flat trays has become the main method for mechanized transplanting of rice in China. However, seedling blocks raised by this method were easily cracked in practice, and this problem can be solved by padding a thin ramie fiber nonwoven film on the bottom surface of seedling tray. This study was conducted to determine the effects of this film on root-zone environment of rice seedlings. The results showed that on the 10^th^ day after sowing, the soil inorganic nitrogen, especially nitrate nitrogen, content in the root-zone of the film treatment were considerably higher than in the no-film treatment, in contrast, the soil organic matter content was lower in the film treatment, and by the 20^th^ day, the gap between treatments was enlarged. After applying the film, the Chao 1 index and Shannon index values for the soil bacterial community diversity decreased, and the rice seedlings were shorter, had higher root/shoot ratios, lower nitrate contents, and higher soluble sugar contents. We conclude that application of the ramie fiber nonwoven film resulted in substantial changes in the soil nutrient and bacterial community in root-zone in a short time, which significantly impacted the growth and development of rice seedlings.

## Introduction

**Figure 1:**
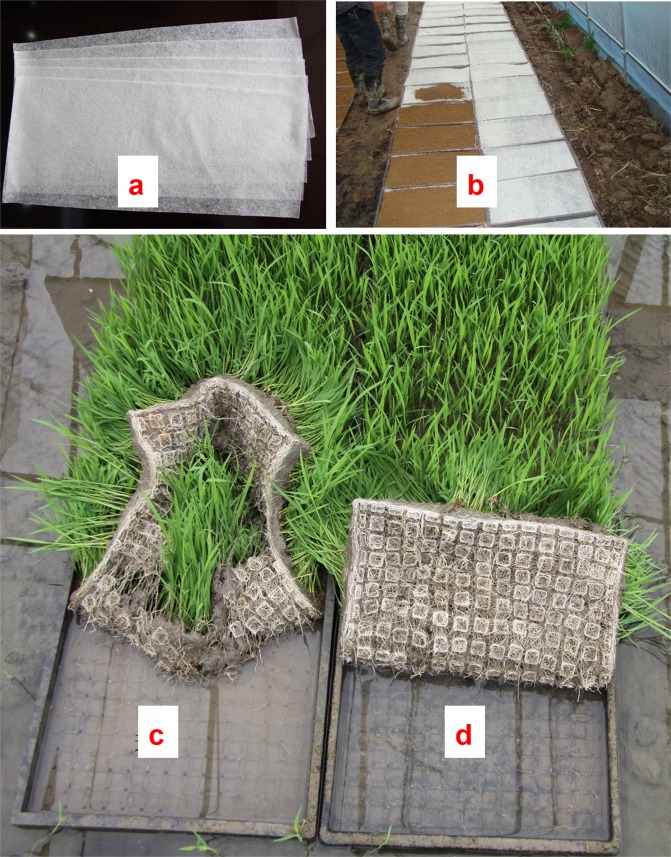
The ramie fiber nonwoven film and its application in raising rice seedlings for mechanized transplanting: (**a**) ramie fiber nonwoven film; (**b**) the use of the film; (**c**) an easily broken rice seedling block (raised without the film); (**d**) a rice seedling block raised with the film.

Rice is a major staple food for about 50% of the world’s population^1^. Rice fields account for more than 12% of global cropland area^2^. China is one of the major rice-producing countries in the world with a rice cultivation area of about 30 million hectares, accounting for approximately 18.6% of the world’s rice field area^3^. With an increasingly scarce rural labor force, raising rice seedlings in flat trays followed by mechanical transplantation has become a prevalent cultivation method to replace hand transplanting in Chinese rice production^4^. However, this method has a serious defect in practice, as the root system of the seedlings was often not sufficiently intertwined by the optimum transplanting date (20–25 days after sowing). Insufficient rooting in the seedling block means the rice seedling blocks easily crack (Fig. 1c), so the efficiency of mechanical transplanting was seriously decreased^5^.

In recent years, An innovative ramie fiber nonwoven film (Fig. 1a) was developed to solve this problem^6^. This film was made of waste fiber from the ramie spinning industry and modified starch, and was constructed using a dry-laid, nonwoven process. Unlike the traditional agricultural nonwoven films that are usually used as mulching materials to cover soil, this film was used to pad the bottom surface of a seedling tray, and was covered by soil (Fig. 1b). The film was quite thin (only about 0.15–0.25 mm); however, previous research^7,8^ showed that the efficiency of machine transplanting was significantly improved by using this film, as it promoted the growth of rice seedling roots and helped to form a strong, not easily broken seedling block (Fig. 1d). Moreover, it can significantly improve rice seedling quality, speed up the emergence of new tillers after transplanting, and increase overall rice yields^9^. Further studies confirmed that the film padded under the soil could increase the oxygen supply to the root-zone of seedlings^10,11^. This increased oxygen supply may directly promote the growth and development of seedlings by promoting root respiration. However, considering the significant effects of soil aeration on the plant rhizosphere (including increased redox potential^12,13^, changed soil nutrient state^14,15^ and microbial activity, abundance, community structure^16–19^, and so on), this film may also cause substantial changes in the root-zone environment of rice seedlings. Identifying these changes not only could help to understand the mechanistic potential of this technology but also may provide new ideas for technological innovation of rice seedling cultivation.

In our study, the soil nutrients, enzyme activities, and bacterial community in the root-zone and rice seedling traits were compared in groups with and without application of the ramie fiber nonwoven film. Our objectives were to (1) examine the effects of using the film padded under the soil on the root-zone environment of rice seedlings and (2) clarify the relationship between these effects and rice seedling traits.

## Results

### Morphological characteristics of rice seedlings

On D10 (the 10^th^ day after sowing), rice seedlings in the M treatment (ramie fiber nonwoven film present) were 12.7% shorter than seedlings in the NM treatment group (not using the film). By D20 (the 20^th^ day after sowing), rice seedlings in the M treatment group were still shorter than the NM treatment seedlings, but the difference was no longer significant (Table 1). On D10, seedlings in the M treatment produced 12.6% less shoot fresh weight, and 17.0% more root fresh weight than NM seedlings. Similarly, on D20, shoot fresh weight of the rice seedlings in the M treatment was 17.6% less than the NM treatment seedlings, while no significant difference in root fresh weight was observed. Differences between M and NM treatment seedlings in biomass dry weight were similar to those in fresh weight (Table 1). Correspondingly, the average root/shoot ratio of rice seedlings in the M treatment was significantly higher than that in the NM treatment group; in terms of fresh weight, the root/shoot ratio increased by 34.5% and 23.1% on D10 and D20, respectively, and in terms of dry weight, the root/shoot ratio increased by 40.6% and 32.0% on D10 and D20, respectively (Table 1).

**Table 1:** Morphological characteristics of rice seedlings on D10 (10^th^ day after sowing) and D20 (20^th^ day after sowing). NM and M represent not padding and padding with ramie fiber nonwoven film on the bottom surface of the seedling tray, respectively.

Sampling date	Treatment	Plant height (cm)	Shoots			Roots			Root/Shoot ratio (fresh weight)	Root/Shoot ratio (dry weight)
			Fresh weight (mg plant^−1^)	Dry weight (mg plant^−1^)	Water csontent (%)	Fresh weight (mg plant^−1^)	Dry weight (mg plant^−1^)	Water content (%)		
D10	NM	7.9 ± 0.4	34.1 ± 1.2	8.2 ± 0.2	76.0% ± 1.0%	10.0 ± 1.8	2.6 ± 0.4	73.7% ± 2.7%	0.29 ± 0.06	0.32 ± 0.04
	M	6.9 ± 0.1	29.8 ± 0.7	7.6 ± 0.3	74.4% ± 1.2%	11.7 ± 1.6	3.4 ± 0.5	70.9% ± 0.2%	0.39 ± 0.05	0.45 ± 0.05
	Paired T-test	DF = 2, t = 4.80 *p* = 0.0407	DF = 2, t = 5.70 *p* = 0.0294	DF = 2, t = 2.83 *p* = 0.1055	DF = 2, t = 1.39 *p* = 0.2999	DF = 2, t = −13.38 *p* = 0.0055	DF = 2, t = −5.69 *p* = 0.0296	DF = 2, t = 1.96 *p* = 0.1885	DF = 2, t = −11.10 *p* = 0.0080	DF = 2, t = −15.58 *p* = 0.0041
D20	NM	11.3 ± 0.8	69.7 ± 9.7	18.6 ± 1.8	73.2% ± 1.2%	18.2 ± 2.9	4.7 ± 0.6	74.2% ± 0.8%	0.26 ± 0.01	0.25 ± 0.01
	M	10.0 ± 0.4	57.4 ± 5.2	16.6 ± 1.1	71.1% ± 0.9%	18.3 ± 2.0	5.4 ± 0.4	70.1% ± 1.5%	0.32 ± 0.01	0.33 ± 0.00
	Paired T-test	DF = 2, t = 3.7 *p* = 0.0634	DF = 2, t = 4.36 *p* = 0.0488	DF = 2, t = 3.80 *p* = 0.0629	DF = 2, t = infty *p* < 0.0001	DF = 2, t = −0.13 *p* = 0.9062	DF = 2, t = −4.55 *p* = 0.0451	DF = 2, t = 13.00 *p* = 0.0059	DF = 2, t = −22.95 *p* = 0.0019	DF = 2, t = −14.42 *p* = 0.0048

### Soluble sugar and nitrate content in rice seedlings

**Figure 2:**
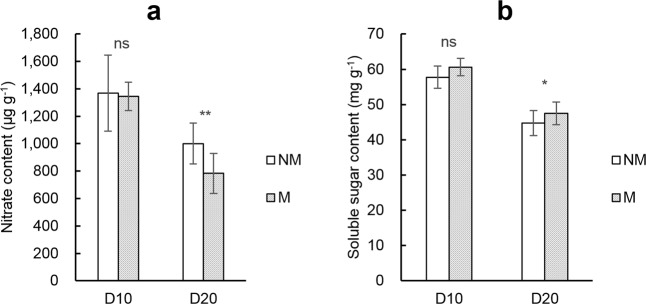
Average nitrate (**a**) and soluble sugar content (**b**) in rice seedling shoots on D10 and D20. NM and M represent not padding and padding with ramie fiber nonwoven films on the bottom surface of the seedling tray, respectively. Error bars represent SE (n = 3). ns, * and ** denote non-significance, significant differences at the 0.05 probability level, and significant differences at the 0.01 probability level between NM and M in each sampling batch (by paired T-test), respectively.

On D10, the average nitrate content in rice seedlings in the M treatment group was 1344.9 μg g^−1^, slightly lower than the NM treatment group (Fig. 2a), the average soluble sugar content was 60.6 mg g^−1^, slightly higher than the NM treatment (Fig. 2b), but none of the differences were statistically significant. By D20, the difference between the treatments was greater, as rice seedlings under the M treatment had an average of 21.7% lower nitrate content and 6.3% higher soluble sugar content than seedlings in the NM treatment.

### Soil pH and EC

**Figure 3:**
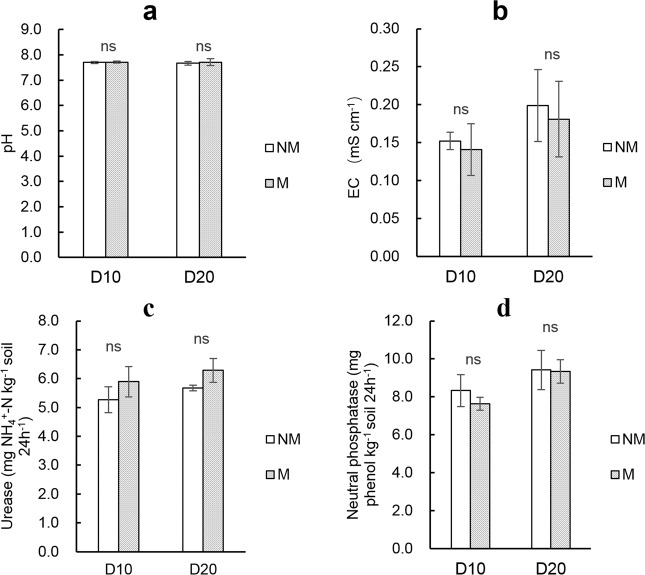
Root-zone soil pH (**a**), EC (**b**), soil urease (**c**) and neutral phosphatase activity (**d**) on D10 and D20. NM and M represent not padding and padding with ramie fiber nonwoven film on the bottom surface of the seedling tray, respectively. Error bars represent SE (n = 3). ns, * and ** denote non-significance, significant difference at the 0.05 probability level, and significant difference at the 0.01 probability level between NM and M in each sampling batch (by paired T-test), respectively.

The application of ramie fiber nonwoven film had no evident effect on root-zone soil pH, which was about 7.7 on both D10 and D20 (Fig. 3a). Soil EC in the M treatment soils was 0.14 and 0.18 mS cm^−1^, on D10 and D20, respectively; and the M group data were slightly lower than the NM treatment mean EC, but the differences were not significant (Fig. 3b).

### Soil nutrients

Compared with the NM treatment, the M treatment had higher soil nitrogen nutrition (Table 2). On D10, mean total nitrogen, nitrate nitrogen, ammonium nitrogen, and alkali-hydrolyzed nitrogen contents in the root-zone soil were 4.9%, 25.4%, 2.3%, and 9.3% higher in the M treatment compared with the NM treatment, but all the differences were not significant except for the alkali-hydrolyzed nitrogen content (*p* = 0.0343). By D20, the gaps between the treatments expanded, and the increase in the M treatment compared to the NM treatment reached 4.7%, 39.3%, 14.6%, 11.5%, respectively, but the only significant difference was in the nitrate nitrogen content (*p* = 0.0148). Compared with the NM treatment, mean available potassium content in root-zone soils in the M treatment increased, while average organic matter and available phosphorus content decreased, but these differences were not significant (*p* > 0.05).

**Table 2:** Nutrients in the root-zone soil on D10 and D20. NM and M represent not padding and padding with ramie fiber nonwoven film on the bottom surface of the seedling tray, respectively.

Sampling date	Treatment	OM (g kg^−1^)	TN (g kg^−1^)	NO_3_^–^N (mg kg^−1^)	NH_4_^+^-N (mg kg^−1^)	AN (mg kg^−1^)	TP (g kg^−1^)	AP (mg kg^−1^)	TK (g kg^−1^)	AK (mg kg^−1^)
D10	NM	23.37 ± 0.35	1.22 ± 0.02	1.26 ± 0.34	4.3 ± 0.4	161.7 ± 8.2	0.51 ± 0.05	29.1 ± 1.9	53.00 ± 1.00	59.7 ± 2.9
	M	22.60 ± 0.20	1.28 ± 0.02	1.58 ± 0.24	4.4 ± 0.6	176.8 ± 3.8	0.53 ± 0.01	28.5 ± 3.2	54.33 ± 1.53	65.7 ± 8.7
	Paired T-test	DF = 2, t = 2.41 *p* = 0.1374	DF = 2, t = −2.43 *p* = 0.1358	DF = 2, t = −2.58 *p* = 0.1229	DF = 2, t = −0.67 *p* = 0.5709	DF = 2, t = −5.26 *p* = 0.0343	DF = 2, t = −0.55 *p* = 0.6349	DF = 2, t = 0.26 *p* = 0.8201	DF = 2, t = −1.11 *p* = 0.3828	DF = 2, t = −1.50 *p* = 0.2724
D20	NM	21.83 ± 0.55	1.29 ± 0.06	1.17 ± 0.13	4.1 ± 0.7	127.2 ± 11.1	0.51 ± 0.03	31.9 ± 3.0	52.67 ± 1.15	53.0 ± 6.9
	M	21.67 ± 0.70	1.35 ± 0.05	1.63 ± 0.03	4.7 ± 0.8	141.8 ± 4.2	0.50 ± 0.01	29.6 ± 2.9	53.67 ± 1.53	58.3 ± 2.5
	Paired T-test	DF = 2, t = 0.27 *p* = 0.8140	DF = 2, t = −2.08 *p* = 0.1732	DF = 2, t = −8.12 *p* = 0.0148	DF = 2, t = −2.77 *p* = 0.1096	DF = 2, t = −3.32 *p* = 0.0800	DF = 2, t = 0.65 *p* = 0.5799	DF = 2, t = 0.80 *p* = 0.5083	DF = 2, t = −1.73 *p* = 0.2254	DF = 2, t = −1.30 *p* = 0.3227

OM, Organic matter. TN, Total nitrogen. AN, Alkali-hydrolyzed nitrogen. TP, Total phosphorous. AP, Available phosphorous. TK, Total potassium. AK, Available potassium.

### Soil enzyme activity

Average soil urease activity in the M treatment was 5.9 and 6.3 mg NH_4_^+^-N kg^−1^ soil 24 h^−1^ on D10 and D20, respectively, and was slightly higher than that under NM, which was 5.3 and 5.7 mg NH_4_^+^-N kg^−1^ soil 24 h^−1^ on D10 and D20, respectively (Fig. 3c). Soil neutral phosphatase activity in the M treatment was 7.6 and 9.3 mg phenol kg^−1^ soil 24 h^−1^ on D10 and D20, respectively, and was slightly lower than that under NM, which was 8.3 and 9.4 mg phenol kg^−1^ soil 24 h^−1^ on D10 and D20, respectively, but these differences were not significant (Fig. 3d).

### Soil bacterial community

**Figure 4:**
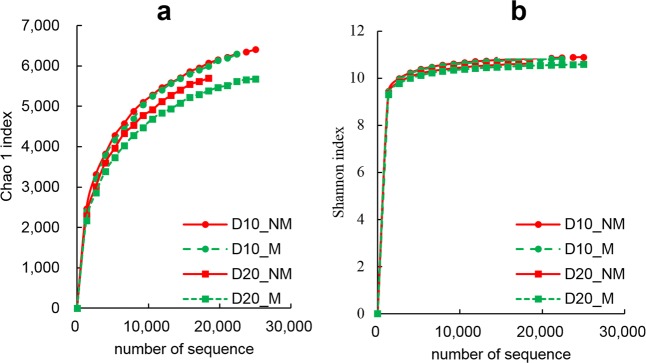
The Chao1 index (**a**) and Shannon index (**b**) of the soil bacterial community in the root-zone on D10 and D20. NM and M represent not padding and padding with ramie fiber nonwoven film on the bottom surface of the seedling tray, respectively.

The Chao1 index of the root-zone soil bacterial community in the M treatment was slightly lower than that in the NM treatment on both D10 and D20 (Fig. 4a). From D10 to D20, the Chao1 index decreased, and compared with the NM group, the decline was greater in the M treatment group, indicating that the soil bacterial diversity in the seedling root-zone decreased as a function of seedling growth time, and the application of the ramie fiber nonwoven film could further reduce it. The Shannon index reflected basically the same trend as the Chao 1 index (Fig. 4b).

**Figure 5:**
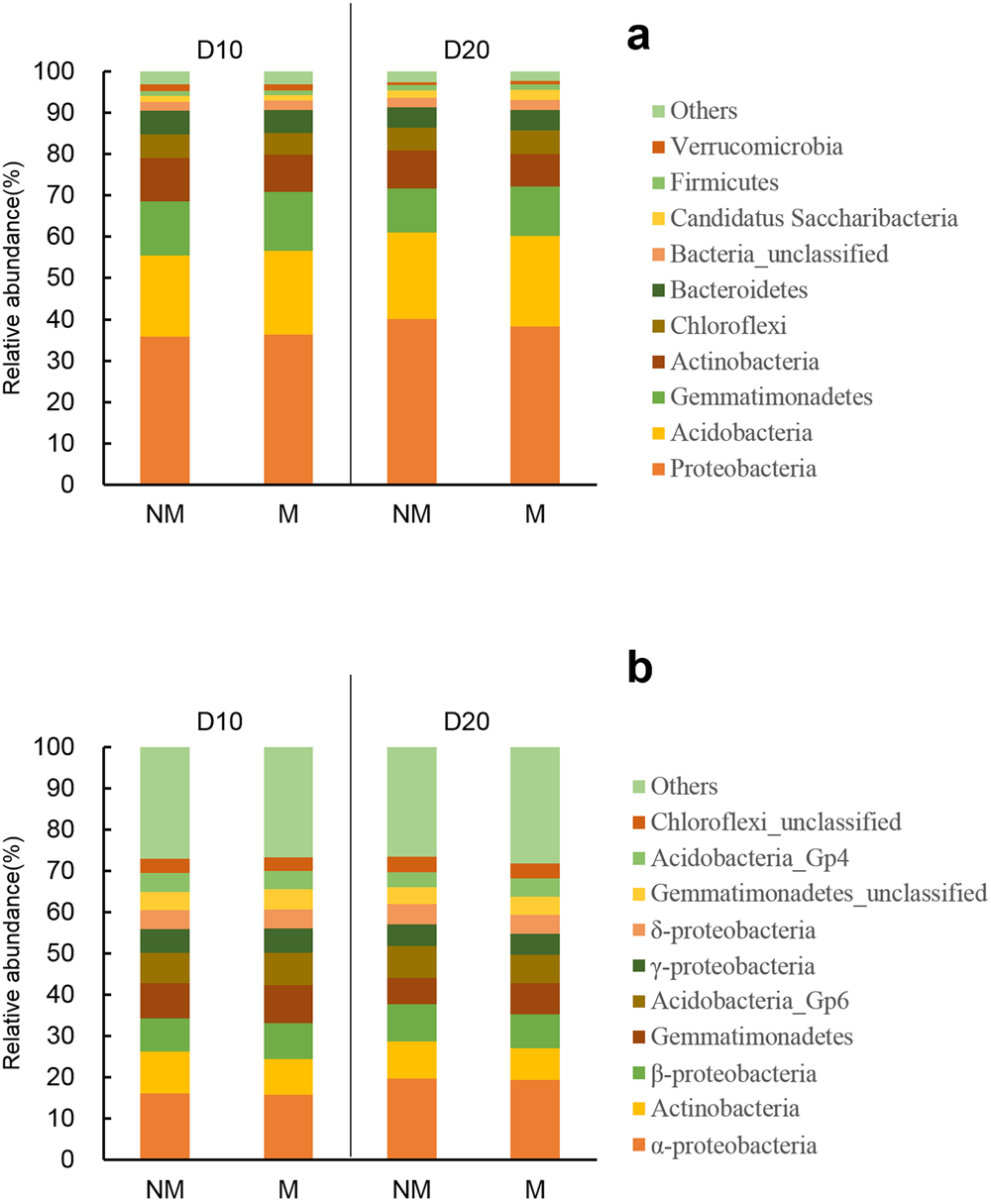
Relative abundance of dominant phyla (**a**) and classes (**b**) of root-zone soil bacterial on D10 and D20. NM and M represent not padding and padding with a ramie fiber nonwoven film on the bottom surface of the seedling tray, respectively.

The highest relative abundance in the soil bacterial community was Proteobacteria (Fig. 5a). In the M treatment it comprised 36.28% and 38.26% of the community on D10 and D20, respectively, and in the NM treatment it comprised 35.78% and 40.08% of the community on D10 and D20, respectively. Followed by Acidobacteria, Gemmatimonadetes, and Actinobacteria, whose total relative abundance when combined with Proteobacteria was close to or over 80% in each treatment. Among different classes of Proteobacteria, α-Proteobacteria always accounted for the highest proportion (about 47%), followed by β-Proteobacteria, γ-Proteobacteria, and δ-Proteobacteria, which each accounted for about 22%, 15%, and 12%, respectively (Fig. 5b).

Compared with NM, the M treatment community showed a trend of increased relative abundance of Acidobacteria and Gemmatimonadetes, but decreased relative abundance of Actinobacteria (Fig. 5a). On D10, the relative abundance of Acidobacteria and Gemmatimonadetes was 20.38% and 14.27%, respectively in the M treatment soils, and 19.75% and 13.00%, respectively in the NM treatment soils. On D20, the relative abundance of Acidobacteria and Gemmatimonadetes was 22.05% and 11.90%, respectively in the M treatment soils, and 20.91% and 10.67%, respectively in the NM treatment soils. The relative abundance of Actinobacteria on D10 and D20 in the M treatment soils was 8.95% and 7.86%, respectively, while the NM treatment soils was 10.57% and 9.15%, respectively.

## Discussion

Our previous studies revealed that the application of the ramie fiber nonwoven film compensated for the rapid oxygen consumption in the bottom soils of seedling trays caused by soil microbial activities, effectively keeping the soil in an oxygen-rich state^10,11^. This study showed that this oxygen-enrichment effect of the ramie fiber nonwoven film further affected soil nutrients, soil microbial composition, and ultimately affected the growth and development of the rice seedlings.

The M treatment had higher inorganic nitrogen content than NM treatment soils (Table 2). There are three possible reasons for this phenomenon. Firstly, increased oxygen supply to the root-zone soil due to the presence of the film enhanced the activity of aerobic microorganisms and consequently accelerated the decomposition of soil organic matter, which was shown by a decrease in organic matter content (Table 2) and, according the finding that excess organic nitrogen could be mineralized into inorganic nitrogen during the process of soil organic matter decomposition when the C/N ratio is less than 25^20–22^ (the initial C/N ratio of the soil in this study was less than 16)), would consequently increase the inorganic nitrogen content in the soil. Secondly, improving root-zone aeration can significantly increase soil enzyme activity, especially soil urease activity^17,23^, which is consistent with the results observed in this study (Fig. 3c). Soil urease is an important regulator of the transformation of organic nitrogen to inorganic nitrogen^24–26^. Therefore, increases in urease activity would improve the mineralization of organic nitrogen, and consequently increase the content of soil inorganic nitrogen. Thirdly, increased oxygen content in the root-zone can also increase the activity of a series of nitrification-related microorganisms such as ammonia-oxidizing bacteria^27^, which could enhance nitrification and promote the transformation of ammonium nitrogen into nitrate nitrogen, and then promote the process of nitrogen mineralization. So, the increased activity of these microorganisms can also contribute to the increase in soil inorganic nitrogen content.

**Figure 6:**
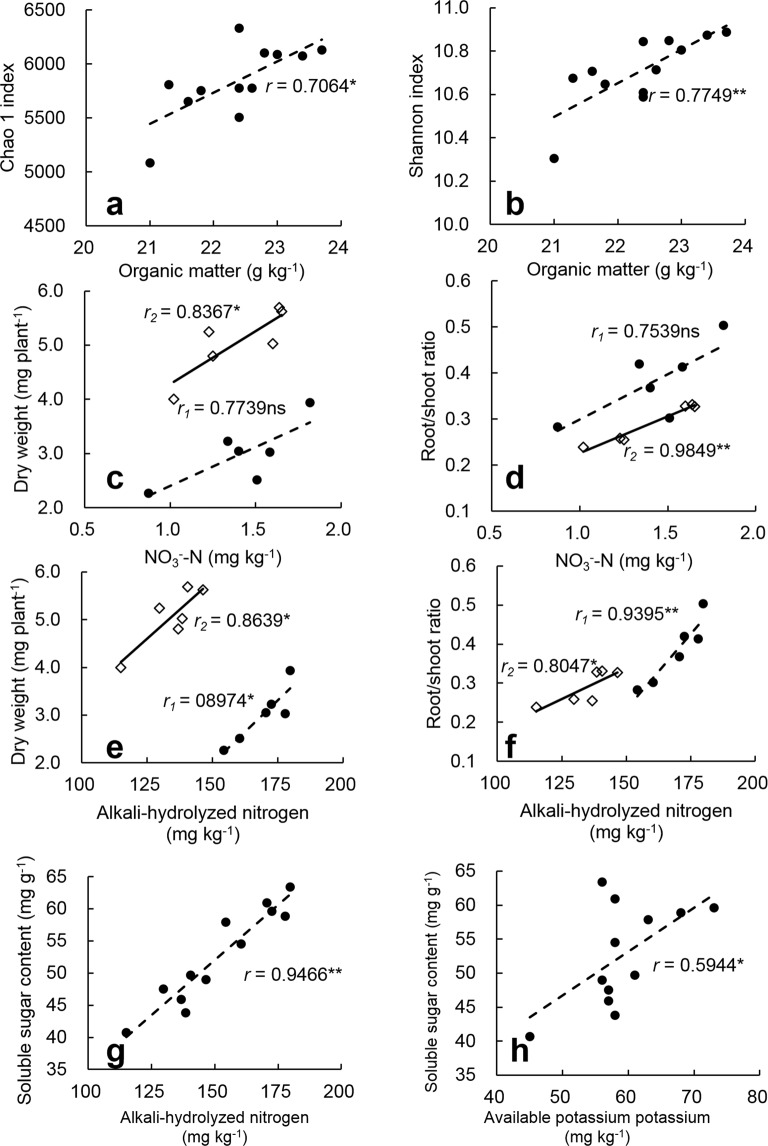
Pearson’s correlations of soil organic matter with the Chao 1 index (**a**, n = 12) and Shannon index (**b**, n = 12); the soil nitrate nitrogen with root weight (**c**) and root/shoot ratio (**d**) of rice seedlings on D10 (dotted line, n = 6) and D20 (solid line, n = 6); the soil alkali-hydrolyzed nitrogen with root weight (**e**) and root/shoot ratio (**f**) of rice seedlings on D10 (dotted line, n = 6) and D20 (solid line, n = 6); and the soil alkali-hydrolyzed nitrogen (**g**, n = 12) and available potassium (**h**, n = 12) with soluble sugar content of rice seedlings. * and ** denote significant differences at the 0.05 probability level and 0.01 probability level, respectively.

Ample evidence has accumulated in recent years that the quantity and quality of soil organic matter can significantly affect the composition of soil microbial community^28–30^, which is often represented by the phenomenon that soils with high organic matter content tend to have higher microbial community diversity^31,32^. In our study, both the Chao 1 index and the Shannon index in the M treatment were lower than that in the NM treatment (Fig. 4a,b), indicating that application of the ramie fiber nonwoven film reduced the soil bacterial diversity in the seedling root-zone. The simultaneous decrease in soil organic matter content (Table 2) and soil bacterial diversity with the application of the ramie fiber nonwoven film obviously accords with above finding, and the significant positive correlations between soil organic matter content and the Chao 1 as well as the Shannon indices (Fig. 6a,b) lend further support for it.

In addition, the relative abundance of Acidobacteria and Gemmatimonadetes in the root-zone soil of seedlings in the M treatment increased (Fig. 5a), which is very similar to the change in the soil bacterial community under dry-raised rice seedling^33^. However, the mechanism and its effects on the growth and development of rice seedling remains unknown.

It is well known that the growth of crop plants, especially the growth of belowground roots, is closely related to the rhizosphere environment; changes in the ecological environment of the rhizosphere will inevitably lead to changes in crop growth factors. After application of the ramie fiber nonwoven film, increases in soil inorganic nitrogen nutrition (especially nitrate nitrogen) may promote root growth of rice seedlings, as confirmed by a large number of studies where nitrate nitrogen stimulated lateral root formation and increased root length^34,35^. This pattern is also supported by the positive correlation of soil nitrate nitrogen and alkali-hydrolyzed nitrogen content with root weight and root/shoot ratio of rice seedling found in this study (Fig. 6c–f). At the same time, the content of other forms of inorganic nitrogen and available potassium in root-zone soils had also increased in the M treatment soils, and positive correlations of the soil alkali-hydrolyzed nitrogen and available potassium with the soluble sugar content of rice seedlings was also observed in this study (Fig. 6g,h). This relationship suggests that these factors together helped to supply enough nutrients to the rice shoot, making the physiological activities of the seedlings more vigorous, and thus, contributed to the accumulation of soluble sugar.

To sum up, application of this film may cause a series of cascading effects. That is, the increasing oxygen supply in the root-zone could promote soil organic matter decomposition, increase soil urease activity as well as aerobic nitrification; and then increased soil nutrition (especially nitrate nitrogen content) and decreased soil organic matter content; consequently improved the nutrient supply of rice seedling and changed the distribution of soil bacteria; finally promoted the growth and development of rice seedling. However, the relation between the change of bacterial community and the growth and development of seedlings is still unknown.

## Conclusions

In a short period of time, the thin ramie fiber nonwoven film laid under the soil in a seedling tray caused substantial changes in the root-zone environment of rice seedlings, including an increase in inorganic nitrogen content and a decrease in soil organic matter content as well as root-zone bacteria diversity, and then led a significant changes in the growth and development of rice seedlings.

## Methods

### Site and soil

The rice seedling experiment was conducted in the experimental field of the Institute of Bast Fiber Crops, Chinese Academy of Agricultural Sciences (N28°12′ E112°44′), Changsha, Hunan, China. The seedlings were raised during one rice season in 2018. Soil taken from the paddy field was crushed and used as seedling soil filled in the cultivation trays. The paddy soil was a Fluvisol (FAO taxonomy) with a clay texture and the following properties: organic matter 24.2 g kg^−1^, total nitrogen 1.53 g kg^−1^, total phosphorus 0.57 g kg^−1^, total potassium 59.0 g kg^−1^, hydrolyzed nitrogen 31.2 mg kg^−1^, available phosphorus 48.4 mg kg^−1^, and available potassium 107.0 mg kg^−1^.

### Materials and treatments

Plastic seedling trays (58 cm × 28 cm × 2.5 cm deep) were used for raising the rice seedlings. This kind of seedling tray has been widely used for raising rice seedlings for machine transplanting in southern China. The ramie fiber nonwoven film was produced by our cooperative enterprise (Haerbin Jingzhu Agricultural Science and Technology Co. Ltd, Haerbin, China). It had an approximate thickness of 0.20 mm and weight of 40 g m^−2^. The experimental rice variety was Huanghua Zhan, which is widely used in one-season rice cultivation in Hunan Province.

Two treatments were used in the experiment: one with the ramie fiber nonwoven film padded on the bottom surface of the seedling tray (M), and the other without ramie fiber nonwoven film padded on the bottom surface of the seedling tray (NM). In order to ensure the consistency of water and soil conditions for the rice seedlings, paired groups consisting of M and NM were arranged in the same seedling tray, that is, half of the bottom surface of seedling tray was covered with ramie fiber nonwoven film, and the other half was not. After padding the film on half of each seedling tray, the trays were filled with seedling soil. Pre-germinated seeds were evenly sown in the seedling soil on April 20, 2018 at a sowing rate of 120 g per tray. After sowing, the seedling trays were placed on the pre-leveled seedling bed in the experimental field, and the soils were kept moist throughout the seedling stage. A total of 15 trays of seedlings were cultivated.

### Sampling

Sampling was carried out on the 10^th^ and 20^th^ days after sowing. At each sampling, three trays of seedlings were randomly selected, and six, 4 × 4 cm seedling blocks were cut from both treatment areas of each tray. Three of the six seedling blocks for each treatment were randomly selected for measurements of rice seedling traits. These were carefully washed to remove the soil. Thirty seedlings with complete root systems were selected from the three seedling blocks, and their plant height was determined. These seedlings were divided into shoots and roots; any surface moisture was removed with paper, and their fresh weight was determined. We measured dry weights after drying at 80 °C to a constant weight, and the dry shoot samples were kept for the determination of soluble sugar and nitrate content. A layer of soil about 1 cm thick at the bottom of the other three seedling blocks was separated and retained as root-zone soil. The visible roots and stones were carefully removed, and the visible film was also removed in the samples taken from the M treatment area. This root-zone soil was mixed and divided into three parts: one part was immediately used for the determination of soil nitrate and ammonium nitrogen content. The second part was air-dried for the determination of soil physiochemical properties, and the third part was kept at −80 °C to determine the composition of the soil bacterial community.

### Soil physiochemical properties

The soluble sugar and nitrate contents were determined by the phenol and salicylic acid methods according to Li^36^. Soil pH and electrical conductivity (EC) were measured in a soil:water mixture (1:5 w/v) using a pH meter (FE20, Mettler-Toledo, Switzerland) and a conductivity meter (DDS-12A, Shanghai Hongyi Instrumentation CO., LTD, China), respectively. We used Bao’s method^37^ for the determination of soil nutrients. Organic matter content (OM) and alkali-hydrolyzed nitrogen (AN) were determined using an outside heating method and an alkaline hydrolysis diffusion method, respectively. Total nitrogen (TN) was determined with an automatic kjeldahl nitrogen determiner (Kjeltec8400, FOSS, Denmark). Soil nitrate nitrogen (NO_3_^–^N) was extracted with a 0.01 mol L^−1^ CaCl_2_ solution and determined by ultraviolet spectrophotometry. Ammonium nitrogen (NH_4_^+^-N) was extracted with a 2 mol·L^−1^ KCl solution and determined using an indigo blue colorimetric method. Total phosphorous (TP) and available phosphorous (AP) were extracted using an HClO_4_-H_2_SO_4_ method and an HCl- H_2_SO_4_ method, respectively, and both were measured using the Vanadium molybdate blue colorimetric method. Total potassium (TK) and available potassium (AK) were extracted by a NaOH molten method and an NH_4_Ac digestion method, respectively, and determined by a flame photometric method. Soil enzyme activity was assayed according to the methods described by Guan^38^. Urease activity was measured by determining the amount of NH_4_^+^ released from a hydrolysis reaction after incubating the samples with urea (10% w/v) for 6 h at 37 °C. Neutral phosphatase activity was estimated by determining the amount of phenol released after incubating the samples with phenyl disodium phosphate (0.5% w/v) for 6 h at 37 °C.

### Soil bacterial community composition

The diversity and composition of the bacterial communities in each sample of root-zone soil were assessed by 16 S rDNA, as described next. Total microbial community DNA was isolated from approximately 0.25 g soil per sample using the E.Z.N.A. ®Soil DNA Kit (D5625, Omega, Inc., USA) according to the manufacturer’s instructions. The reagent was designed to uncover DNA from trace amounts of the sample and has been shown to be effective for the DNA preparation of most bacteria. The V3-V4 region of the 16 S rDNA gene was amplified with slightly modified versions of primers 338 F (5′-ACTCCTACGGGAGGCAGCAG-3′) and 806 R (5'-GGACTACHVGGGTWTCTAAT-3′)^39^. The 5′-ends of the primers were tagged with sample-specific barcodes and universal sequencing primers. PCR amplification was performed in a total volume of 25 µL reaction mixture containing 25 ng of template DNA, 12.5 µL PCR Premix, 2.5 µL of each primer, and PCR-grade water to adjust the volume. The PCR conditions to amplify the prokaryotic 16 S fragments consisted of an initial denaturation at 98 °C for 30 seconds, 35 cycles of denaturation at 98 °C for 10 seconds, annealing at 54 °C for 30 seconds, a primary extension at 72 °C for 45 seconds, and then a final extension at 72 °C for 10 minutes. The PCR products were confirmed with 2% agarose gel electrophoresis, purified by AMPure XT beads (Beckman Coulter Genomics, Danvers, MA, USA), and quantified by Qubit (Invitrogen, USA). The amplicon pools were prepared for sequencing, and the size and quantity of the amplicon library were assessed on an Agilent 2100 Bioanalyzer (Agilent, USA), and with the Library Quantification Kit for Illumina (Kapa Biosciences, Woburn, MA, USA), respectively. A PhiX Control library (v3) (Illumina) was combined with the amplicon library (expected at 30%). Paired-end reads were assigned to samples based on their unique barcode, and then truncated by cutting off the barcode and primer sequence. Paired-end reads were merged using FLASH. Quality filtering of the raw tags was performed under specific filtering conditions to obtain the high-quality clean tags according to FastQC (V 0.10.1). Chimeric sequences were filtered using, and sequences with ≥97% similarity were assigned to the same operational taxonomic units (OTUs) using the VerSearch software (v2.3.4). Representative sequences were chosen for each OTU, and taxonomic data were then assigned to each representative sequence using the RDP (Ribosomal Database Project) classifier. OTU abundance information was normalized using a standard of the sequence number corresponding to the sample with the least sequences. Alpha diversity was applied in analyzing the complexity of species diversity for a sample through 2 indices: Chao1 and Shannon. All of our indices statistics were calculated with QIIME (Version 1.8.0).

### Statistical analysis

The data were analyzed using SAS 8.2. Treatment means were compared using paired T-tests. Pearson’s correlation analyses were conducted to investigate the relationships among soil physicochemical properties, soil microbial community diversity and rice seedling traits.

## Data Availability

All data generated or analysed during this study are included in the article.
