# Author Correction: Effects of applying ramie fiber nonwoven films on root-zone soil nutrient and bacterial community of rice seedlings for mechanical transplanting

**DOI:** 10.1038/s41598-020-64240-9

**Published:** 2020-04-30

**Authors:** Wanlai Zhou, Jing Chen, Zhiyong Qi, Chaoyun Wang, Zhijian Tan, Hongying Wang, Zhenxie Yi

**Affiliations:** 1 College of Agronomy, Hunan Agricultural University, Changsha, 410128 China; 2 Institute of Bast Fiber Crops, Chinese Academy of Agricultural Sciences, Changsha, 410205 China; 3 Institute of Urban Agriculture, Chinese Academy of Agricultural Sciences, Chengdu, 610213 China

Correction to: *Scientific Reports* 10.1038/s41598-020-60434-3, published online 26 February 2020

In the original version of this Article, Chaoyun Wang was omitted as a corresponding author. Correspondence and request for materials should be addressed to ibfcwcy@139.com. This error has now been corrected in the HTML and PDF versions of the Article.

